# Pharmacogenomics to Predict Tumor Therapy Response: A Focus on ATP-Binding Cassette Transporters and Cytochromes P450

**DOI:** 10.3390/jpm10030108

**Published:** 2020-08-28

**Authors:** Viktor Hlaváč, Petr Holý, Pavel Souček

**Affiliations:** 1 Toxicogenomics Unit, National Institute of Public Health, 100 00 Prague, Czech Republic; petr.holy@szu.cz (P.H.); pavel.soucek@szu.cz (P.S.); 2 Laboratory of Pharmacogenomics, Biomedical Center, Faculty of Medicine in Pilsen, Charles University, 306 05 Pilsen, Czech Republic; 3 Third Faculty of Medicine, Charles University, 100 00 Prague, Czech Republic

**Keywords:** cancer therapy, response, pharmacogenomics, ATP-binding cassette transporters, cytochromes P450, omics, personalized medicine

## Abstract

Pharmacogenomics is an evolving tool of precision medicine. Recently, due to the introduction of next-generation sequencing and projects generating “Big Data”, a plethora of new genetic variants in pharmacogenes have been discovered. Cancer resistance is a major complication often preventing successful anticancer treatments. Pharmacogenomics of both somatic mutations in tumor cells and germline variants may help optimize targeted treatments and improve the response to conventional oncological therapy. In addition, integrative approaches combining copy number variations and long noncoding RNA profiling with germline and somatic variations seem to be a promising approach as well. In pharmacology, expression and enzyme activity are traditionally the more studied aspects of ATP-binding cassette transporters and cytochromes P450. In this review, we briefly introduce the field of pharmacogenomics and the advancements driven by next-generation sequencing and outline the possible roles of genetic variation in the two large pharmacogene superfamilies. Although the evidence needs further substantiation, somatic and copy number variants as well as rare variants and common polymorphisms in these genes could all affect response to cancer therapy. Regulation by long noncoding RNAs has also been shown to play a role. However, in all these areas, more comprehensive studies on larger sets of patients are needed.

## 1. Introduction

Pharmacogenetics is not a novel term. It was first used in 1959 by a German geneticist Friedrich Vogel. The term pharmacogenetics was understood as “phenotypic variation in metabolism and response to drugs” [1]. Later on, advances in analytical methods and gene cloning resulted in an improved understanding of the genetic basis of this variation. The cloning and sequencing of the entire human genome in the late 1990s brought with them another term—pharmacogenomics. These two terms are now used interchangeably, although the term pharmacogenomics is broader, also comprising the development of new drugs targeting specific disease-causing genes [2].

Pharmacogenetics has always been an important part of personalized therapy. The absorption, distribution, metabolism, and excretion (ADME) of drugs, pharmacokinetics (PK) and pharmacodynamics (PD) affect drug efficacy and toxicity and can thereby cause adverse drug reactions (ADRs) or therapy failure. All of these factors are influenced by inter-individual variations largely dictated by genotype, and thus pharmacogenetics has also become important in the clinical setting [3].

Initially, only hereditary germline variation was the primary focus of pharmacogenetics. However, after breakthroughs in genotyping techniques and sequencing, such as capillary sequencing, cancer genetics/genomics became a viable direction for research [4]. Tumor somatic mutations were revealed to be an important aspect of cancer treatment, allowing for the design of a number of new drugs targeting specific gene mutations [5]. After commercial use of next-generation sequencing (NGS) began in 2005, many new variants were discovered in known pharmacogenes. Pharmacogenetics/pharmacogenomics rapidly expanded as new “Big Data” sequencing projects (e.g., 1000 Genomes Project) were established. It was soon evident that the number of variants in pharmacogenes would be much greater than previously thought. For example, the number of variants in the cytochrome P450 (CYP) gene superfamily in the European population was calculated to be 3.4 × 10^4^ [6]. Rare variants (minor allele frequency (MAF) < 1%) are also an important part of pharmacogenetics and constitute the majority of variant alleles in pharmacogenes. Recent studies on Exome Aggregation Consortium (ExAC) and the 1000 Genomes Project corroborate the importance of rare genetic variants in the pharmacogenetic prediction of drug response [7,8].

The number of publications about pharmacogenetics/pharmacogenomics is growing rapidly. While in 2000 only 57 articles published that year in the PubMed database [9] mentioned the term “pharmacogen* AND cancer“—this number rose to 365 in 2010 and almost 700 articles came out in 2019. Therefore, summarizing all known pharmacogenes that could be of interest in cancer therapy is beyond the scope of this review. Advancements in personalized therapy and a comparison between the germline and somatic aspects of pharmacogenetics were summarized by Hertz and McLeod [10], McLeod [11] and recently by Hyman et al. [5]. 

Common pharmacogenes predominantly consist of phase I and phase II biotransformation enzymes, cytochrome reductases, membrane transporters, and nuclear receptors. The genetic variability in these genes can modulate ADME and may cause ADRs or changes in drug efficacy [8]. As a result, exposure of cancer cells to a lower therapeutic dose can lead to resistance to therapy. Cancer chemoresistance and suboptimal responses to therapy have been a major obstacle for successful cancer treatments and can stem from a number of factors. 

**Figure 1:**
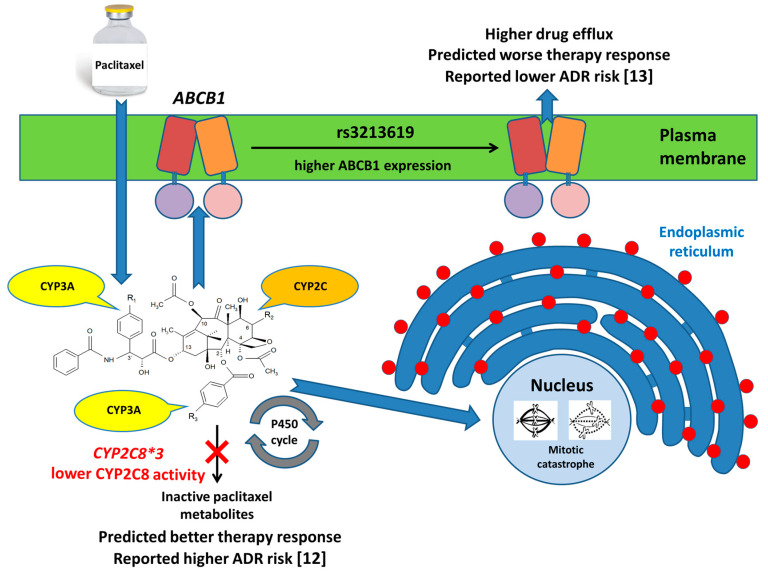
Major functional aspects of ATP-binding cassette (ABC)/cytochrome P450 (CYP) in drug responses with the presumed role of their genetic variability.

ATP-binding cassette (ABC) transporters may induce drug resistance by transporting drugs across cellular membranes. CYP monooxygenases metabolize a broad range of drugs or activate prodrugs. Together, these groups represent a significant portion of known pharmacogenes. The concept of genetic variants in ABCs and CYPs affecting the response of a cancer cell to an anticancer drug (e.g., paclitaxel) is presented in Figure 1. This article will focus solely on the genomic variation in ABC transporters and CYPs and their possible roles in tumor therapy responses and will outline the most recent findings, summarizing the perspectives of their potential use in personalized therapies on germline, somatic and copy number levels. In addition to genomics, an emerging direction of studying noncoding regulatory molecules will be discussed.

The cartoon displays the roles of ABC and CYP pharmacogenomics in anticancer treatments. Paclitaxel serves as an example. P450 variant CYP2C8*3 lowers the activity of the enzyme, resulting in more paclitaxel in the nucleus with a higher ability to cause mitotic catastrophe. CYP2C8*3 was found to be associated with an increased risk of taxane-related sensory neuropathy [12]. Simultaneously, paclitaxel might be the subject of efflux outside of the cell, e.g., by p-glycoproteins coded by *ABCB1*, lowering its therapeutic efficacy. Variant rs3213619 could mean a higher paclitaxel efflux and worse predicted prognosis. On the other hand, variant rs3213619 was associated with a decreased risk of taxane-related sensory neuropathy [13].

## 2. Germline Variants in ABC Transporters and CYP Genes

Germline single nucleotide variants in CYPs may modulate the metabolic activity in the liver and thus affect the prodrug activation or detoxification of active drugs in the organism. Secondly, hereditary genetic variability in membrane efflux transporters may alter the effective concentration of drugs in tumor cells. Furthermore, heterozygotic alterations may couple with a loss in the heterozygosity of the second allele in tumor cells or epigenetic dysregulation and impair cellular transport. Thus, assessing hereditary genetic information before prescribing anticancer drugs might be beneficial for patients’ life expectancy and quality of life and for health care system sustainability.

According to the U.S. Food and Drug Administration (FDA) Table of Pharmacogenomic Biomarkers in Drug Labeling [14], several diagnostic tests are mandatory before the prescription of anticancer drugs. Most of these drugs are tyrosine kinase inhibitors (TKIs) [15]. Focusing on germline variants, among the anticancer drugs found in drug labels are the EGFR inhibitor gefitinib, FGRF inhibitor erdafitinib, PARP inhibitor rucaparib, and tamoxifen, a selective modulator of the estrogen receptor [14]. European Medicines Agency (EMA) has a drug label also for the TKI sunitinib [16], but it is only related to drug–drug interactions with *CYP3A4* (avoidance of *CYP3A4* inhibitors), not its genetics. FDA drug labels for these compounds are described in Table 1. In the FDA drug labels, no dose recommendations are applicable but “The exposure of erdafitinib is predicted to be 50% higher in subjects with the CYP2C9*3/*3 genotype, estimated to be present in 0.4% to 3% of the population among various ethnic groups”. The rest is limited to the recommendations for the monitoring of ADR in patients with a poor metabolizer genotype (gefitinib) or information about metabolizer phenotypes (rucaparib and tamoxifen).

In the Pharmacogenomics Knowledgebase [17] multiple members of ABC and CYP superfamilies are indicated as “Very Important Pharmacogenes” (VIP) [18]. These are: *ABCB1*, *ABCG2*, *CFTR*, *CYP1A2*, *CYP2A6*, *CYP2B6*, *CYP2C8*, *CYP2C9*, *CYP2C19*, *CYP2D6*, *CYP2E1*, *CYP2J2*, *CYP3A4*, *CYP3A5*, and *CYP4F2*. However, most of the clinical associations are not related to anticancer drugs. For these, a high level of evidence is fulfilled only by tamoxifen dosage adjustment for *CYP2D6* haplotypes (CYP2D6*1, CYP2D6*10, CYP2D6*3, CYP2D6*4, CYP2D6*41, CYP2D6*5, and CYP2D6*6) in breast cancer. Patients with the CYP2D6*1/*1 genotype, i.e., normal metabolizers, have an increased metabolism of tamoxifen. These patients show increased concentrations of tamoxifen’s active metabolite endoxifen, resulting in decreased cancer recurrence and improved disease-free survival during adjuvant tamoxifen treatment. Poor metabolizers carrying no functional alleles are recommended to use an alternate hormonal therapy, such as aromatase inhibitor anastrozole [19,20,21,22]. A moderate level of evidence (i.e., results are replicated, but some studies are not significant, and/or the effect size may be small) exists also for the variant rs3892097 in *CYP2D6* (CYP2D6*4). Patients treated with tamoxifen who have the AA genotype, i.e., poor metabolizers, show an increased risk of relapse compared with patients with the GA or GG genotypes [18]. Finally, a well described variant rs4646 in the aromatase gene *CYP19A1* is associated with the efficacy of anastrozole, letrozole, or tamoxifen in breast cancer patients [23,24]. Other studies concerning therapy efficacy or metabolism/PK which are described in the section of clinical annotations in PharmGKB have low (nonreplicated study or multiple studies lacking clear evidence of an association) or preliminary (in vitro, nonsignificant study or case-report) levels of evidence. Studies with levels of evidence from 1 to 3 (high–low) are summarized in Table 2. The studies with low levels of evidence will not be discussed further for the sake of brevity.

The role of rare germline variants in pharmacogenetics was recently underlined by Kozyra et al. [7]. Their finding was further confirmed by studies on ExAC data. Rare genetic variants constituted the vast majority of variants in pharmacogenes (98.5% out of 69,923 variants in 208 pharmacogenes or 97.5% out of 61,134 functional variants in 806 pharmacogenes) [8,25]. Rare pharmacogenetic variants were also highly enriched in those that were predicted to be functional alterations. In addition, rare variants were abundant in the genes of the irinotecan (topoisomerase inhibitor prodrug) pathway, accounting for 100% of variants in *ABCB1*, *ABCC2*, and *ABCG2*. Rare variants also represented more than 40% of variants in *ABCC1* and *CYP3A4* and to a lesser extent in *CYP3A5* [8]. Quite recently, based on the data from The Cancer Genome Atlas (TCGA), it was discovered that a variant burden in *ABCC1* substantially predicts disease-free survival in breast cancer patients and that this significance was even stronger in cyclophosphamide- and doxorubicin-treated subgroups [26]. Therefore, rare variants account for a substantial part of drug metabolism and may become an important factor in genotyping-based predictions of drug response.

In summary, germline variants in many of the VIPs of CYP and ABC superfamilies as well as in other genes (*ABCB4, ABCC1-C4, CYP1A1*, and *CYP1B1*) are associated with drug response in cancer. A recent study brought evidence that the germline genetic component was at least as important in the prediction of drug sensitivity as somatic mutations in 993 cell lines treated with 265 drugs [27]. Germline variants are thus a relevant part of pharmacogenetic studies in cancer therapy.

**Table 2:** Pharmacogenomics Knowledgebase (PharmGKB) clinical annotations of ABC and CYP genes.

Gene ^1^	Variant	Drug	Type	Disease	Reference
***ABCB1***	**rs10276036, rs2032582, rs1128503, rs1045642, rs2229109, rs4148737**	**platinum compounds, cyclophosphamide, anthracyclines, methotrexate, vincristine, paclitaxel, sunitinib, imatinib, cytarabine, lenalidomide, 5-FU, tamoxifen**	**efficacy**	**OSRC, BC, OVC, RCC, AML, multiple myeloma, CML, NSCLC, CRC, EC**	[28,29,30,31,32,33,34,35,36,37,38,39,40,41,42,43,44,45,46,47,48,49,50,51,52,53,54]
*ABCB4*	rs1202283	imatinib	efficacy	GIST	[55]
*ABCC1*	rs6498588	irinotecan	PK	CRC	[56]
*ABCC2*	rs2273697, rs3740065	Imatinib, tamoxifen	efficacy	GIST	[54,55,57]
*ABCC3*	rs4148416, rs9895420	cisplatin, methotrexate	efficacy	OSRC, ALL	[28,58]
*ABCC4*	rs9561765, rs16950650	imatinib, cisplatin	efficacy	GIST, SCLC	[59,60]
***ABCG2***	**rs2231142, rs2725252, rs7699188, rs13120400, rs12505410**	**capecitabine, 5-FU, leukovorin, oxaliplatin, imatinib, gemcitabine, irinotecan**	**efficacy**	**CRC, AML, NSCLC, CML**	[61,62,63,64]
*CYP1A1*	rs1048943	capecitabine, docetaxel	efficacy	BC	[65]
***CYP1A2***	**rs762551**	**imatinib**	**dosage**	**GIST**	[66]
*CYP1B1*	rs1056836	5-FU, anthracycline, cyclophosphamide	efficacy	BC	[67]
***CYP2A6***	*1A, *4A	**tegafur**	**PK**	**healthy liver**	[68]
***CYP2B6***	rs12721655, rs3745274, *1, *6	**cyclophosphamide, doxorubicin, imatinib**	**efficacy**	**BC, CML, CLL**	[43,69,70]
***CYP2C19***	rs4244285, *1/ *17/*2	**cyclophosphamide, doxorubicin, tamoxifen**	**efficacy**	**BC**	[43,71]
***CYP2D6***	*1/*10/*3/*4/*41/*5/*6 ^2^, rs3892097 ^3^, *1/*10/*114/*2/*3/*4/*41/*4xN/*5/*6	**tamoxifen, gefitinib**	**efficacy, PK**	**NSCLC, BC**	[19,20,21,22,72,73,74,75,76,77,78]
***CYP2E1***	**rs6413432, rs2070676**	**cisplatin, cyclophosphamide**	**efficacy**	**OVC**	[79]
***CYP3A4***	**rs12721627**	**paclitaxel**	**dosage**	**NSCLC**	[80]
***CYP3A5***	**rs776746**	**imatinib**	**PK**	**CML**	[50]
***CYP19A1***	**rs4646 ^3^**	**anastrozole, letrozole, tamoxifen**	**efficacy**	**BC**	[23,24]

^1^ Very important pharmacogenes (VIP) are depicted in bold. ^2^ Level 1A association. ^3^ Level 2A/B associations (The rest of the associations are level 3, i.e., low level of evidence). Abbreviations: 5-FU—5-fluorouracil; ALL—acute lymphoblastic leukemia; AML—acute myeloid leukemia; BC—breast cancer; CLL—chronic lymphocytic leukemia; CML—chronic myeloid leukemia; CRC—colorectal cancer; EC—esophageal cancer; GIST—gastrointestinal stromal tumor; NSCLC—non-small cell lung cancer; OSRC—osteogenic sarcoma; OVC—ovarian cancer; PK—pharmacokinetics; RCC—renal cell carcinoma; SCLC—small cell lung cancer.

## 3. Somatic Variants in ABCs and CYPs in Solid Tumors

As outlined above, a mutation in a gene coding for a protein that metabolizes/transports a therapeutic drug could, in principle, affect the function of the protein, and thus lead to a change in the response of the cancer cell to treatment. However, while the area of germline variation and gene expression of ABC transporters and CYPs has been a major research focus for decades, only recently have somatic variations been given similar attention, owing to the advancements in knowledge and technology. The level of evidence (or lack thereof) is in stark contrast here.

While a number of pharmacogenes have been approved by the FDA as biomarkers and are used in drug labeling [14], none of the genes are ABC transporters and only three CYPs are on the list. More importantly, only the germline variation in population is considered, not variation due to somatic mutations in tumors, despite the reasonable expectation that it is indeed somatic, rather than germline, variation acquired by cancer cells that is in most cases behind the poor response of a tumor to therapy.

**Figure 2:**
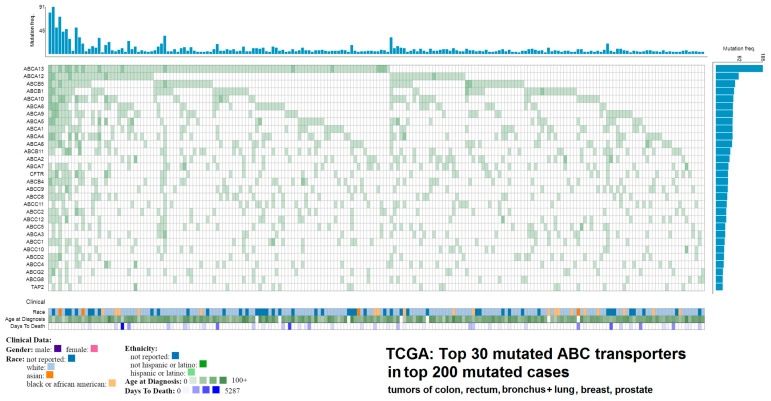
Top 30 mutated ATP-binding cassette transporters in top 200 mutated cases in The Cancer Genome Atlas (TCGA) across the four most common cancers types. The results shown here are in whole based upon data generated by the TCGA Research Network: [81].

**Figure 3:**
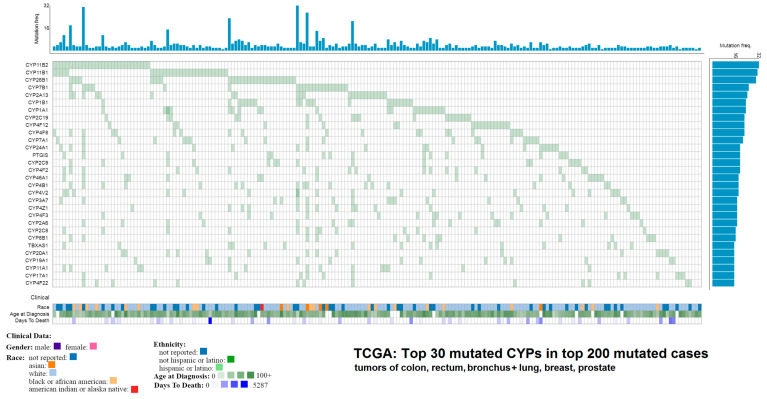
Top 30 mutated cytochromes P450 in top 200 mutated cases in TCGA across the four most common cancers types. The results shown here are in whole based upon data generated by the TCGA Research Network: [81].

In TCGA (data release 23.0) [81], which collects data from dozens of large-scale genomic projects and currently contains mutation data from more than 83 thousand cases, including 67 primary tumor sites, there are in total 17,329 mutations indexed for 48 functional protein-coding ABC transporter genes and 9789 mutations for 57 CYPs. These variants can be further filtered by their Variant Effect Predictor (VEP), Sorting Intolerant From Tolerant (SIFT) of Polymorphism Phenotyping (PolyPhen) scores, representing their probable impact on the phenotype. For example, in ABC transporters, 1506 mutations in 48 genes are classified as having high impact according to the VEP score, meaning they cause a frameshift, stop gain, stop loss, start loss or a splice alteration. Hypothetically, any one of these variants could significantly affect the resulting protein and thus modulate its function. Since many ABCs are known to transport drugs out of cells and lower their efficacy, damaging the function of these proteins could lead to a higher sensitivity to therapeutics [82]. The function of CYPs, well-known drug metabolizers (794 high impact mutations in 57 genes) could be possibly altered in an analogous fashion. However, practically no high-level experimental studies exist that clearly confirm or disprove any potential roles of these variants in cancer therapy. The top 30 mutated (any predicted impact, incl. missense variants) ABC transporters (Figure 2) and CYPs (Figure 3) in TGCA in the top 200 mutated cases across the four most common cancers (colorectal, lung and bronchus, breast, and prostate) are presented for an overview.

A similar situation exists in the Catalogue of Somatic Mutations in Cancer (COSMIC) database (v91), a massive database that consists of more than 6 million coding mutations measured in 1.4 million tumor samples (including those in TCGA), manually curated from over 26 thousand publications [83]. In their list of genes whose somatic mutations have been implicated in drug resistance, no ABC transporters or CYPs are listed. Interestingly, *CYP2C8* is included in their Cancer Gene Census [84], a list of genes which contain mutations causally implicated in cancer, with the level of evidence (Tier 2) described as “strong indications of a role in cancer, but with less extensive available evidence (than Tier 1)”. This finding was based on a 2018 study suggesting that *CYP2C8* is an active driver of cancer after panel sequencing tumor samples from 273 patients with colorectal cancer (CRC) and an analysis of the somatic variants [85].

Mucinous CRC is known for a poorer response to chemotherapy and a worse prognosis than other types of CRC, owing largely to a higher rate of resistance [86]. Reynolds et al. [87] recently analyzed TCGA data for 67 mucinous CRC and compared them to 456 samples of non-mucinous CRC. They looked for associations of 26 pharmacogenes with response to irinotecan, oxaliplatin and 5-fluorouracil (5-FU). The somatic mutation rate between the two cohorts was compared and two genes were identified as having statistically significantly different rates: *ABCB1* (*p* = 0.042), and *ABCG2* (*p* = 0.010). The mutation rate of these two genes associated with resistance to irinotecan. The mutations did not lead to a statistically significant difference in the expression of corresponding proteins; however, the authors postulated that these mutations result in overactive proteins that more effectively export irinotecan out of the cell, lowering its efficacy [87].

While the ABC and CYP superfamilies are well established in pharmacology and oncology as influencers of drug responses, we are yet to see the vast data on their somatic genetic variation in tumors being utilized in a fashion similar to how gene expression and germline variation data are being used already in the clinic. This disparity is striking, considering the number of mutations in these important pharmacogenes that arise in tumors.

## 4. Copy Number Variants in ABCs and CYPs

Structural variation, including alterations in gene copy numbers, is a key mutational process in cancer development and progression [88,89]. In addition to their potential predictive value in terms of cancer onset, copy number variations (CNVs) can also help point out tumors with a poor prognosis and even provide therapeutically useful information [90].

The fact that germline CNVs are frequently observed in pharmacogenes (PharmGKB) was recently demonstrated by a retrospective study on data from the 1000 Genomes Project and ExAC. CNVs that are present in 97% of common pharmacogenes accounted for 5% of all the loss-of-function alleles in 42% of the studied pharmacogenes. Novel deletions were present with particularly high MAF in *CYP2C19* and *CYP4F2* [91]. The authors recommend the utilization of CNV detection assays for the testing of relevant genes in appropriate populations. Therefore, CNVs are another important source of drug response variation.

In oncology, CNVs in *CYP2D6* are among the most frequently studied structural variations in connection with efficacy and ADR of tamoxifen treatment in breast cancer patients. A recent study reported that about 26% of Ethiopian breast cancer patients had germline *CYP2D6* amplifications, predicting a significantly increased plasma concentration of endoxifen, the active tamoxifen metabolite, in most cases [92]. This study confirmed previous recommendations of the Clinical Pharmacogenetics Implementation Consortium (CPIC) suggesting that patients with gene amplifications should avoid moderate or strong *CYP2D6* inhibitors [20], but a population-specific context warrants caution as even within Europe the inter-population variability in *CYP2D6* genetics is high [93]. Despite notable progress in this area, some technical challenges persist, e.g., problems with an accurate assignment of the amplified allele in CNV heterozygotes [94,95] which have to be carefully standardized which includes the increasingly popular massive parallel sequencing methods [96]. In addition to the previously mentioned importance of germline single nucleotide polymorphisms in *CYP19A1* for the treatment of breast cancer patients with aromatase inhibitors, a recent study observed that more than 20% of patients treated with inhibitors acquired *CYP19A1* amplifications [97], suggesting a counter-attack of tumor cells against this therapy in a significant portion of patients and a need for further optimization.

In parallel with the FDA and EMA recommendations on the testing of germline polymorphisms, CNVs in *CYP2A6*, *CYP2D6* and *CYP2E1* (and *GSTM1* or *GSTT1*) were found overrepresented among good responders to therapy with the TKIs imatinib, dasatinib or nilotinib in a small-scale study of patients with chronic myeloid leukemia [98]. However, this potentially useful observation for the optimization of targeted therapy awaits replication.

Among drug efflux transporters, few reports about the role of their CNVs in cancer are available. Recently reviewed information [99] suggested that an *ABCB1* locus including another transporter *ABCB4* is frequently amplified in cancer and mainly in tumors or in vitro models with induced drug resistance [100]. This could present an additional opportunity for intervention (in addition to clinically tested inhibitors) against hard to treat tumors. Outside of numerous studies on *ABCB1* in drug resistance, *ABCC1* was also found to be amplified in drug resistant in vitro models [101] and may be considered a target for future studies in this area. 

A study of 128 discordant sibling pairs, validated in 1048 Chinese Han subjects, revealed an association of CNVs at 13q32.1, where *ABCC4* is located, with an increased risk of esophageal squamous cell carcinoma [102], suggesting that CNVs should be investigated also in less studied transporters. The relevance of this observation to cancer progression or therapy awaits further elaboration.

The data paucity can be demonstrated by the analysis of publicly available databases (Table 3), where large germline CNVs (larger than 1 kb) can be identified only in *CYP2D6, CYP4A11, CYP17A1, ABCD3*, and *ABCE1* (the Genome Aggregation Database—gnomAD [103]). None of these genes are either recognized as disease causing (in Database of Chromosomal Imbalance and Phenotype in Humans Using Ensembl Resources —DECIPHER [104]) or have pathogenic large CNVs of clinical significance in the ClinVar database [105]. Moreover, all germline CNVs, except MCNV_22_1026 in *CYP2D6*, are rather rare CNVs, with frequencies smaller than one allele per thousand (Table 3). Complementary analysis of somatic CNVs using the COSMIC database v91 (all databases accessed on 5 May 2020) demonstrates that highest counts of CNVs among all available cancers were observed in the less studied genes *CYP11B1*, *CYP11B2*, *CYP4V2*, and *ABCA5-A12*. In contrast to the pharmacogenes presented in Table 1, somatic CNVs were listed only in *CYP2C9* at approximately half the frequency compared to *CYP11B1/2*. The same basically applies to ABC transporters listed in Table 2, except for *ABCB1*, *ABCB4* and *ABCC2*. However, unlike the CYPs, the length of individual ABC genes markedly differs within the superfamily (e.g., the length of *ABCC2* is 70kb while the length of *ABCB1* is 210kb), a fact that calls for cautious interpretation in this particular case.

From the above information, it is obvious that CNVs represent valuable information in addition to single nucleotide polymorphisms and rare variants, and, after a thorough standardization of methodology for their assessment and interpretation, should supplement a predictive, diagnostic, and prognostic exploitation of gene panels in oncology and therapeutics.

## 5. Long Noncoding RNA Regulation

A recently discovered source of inter-individual differences in pharmacogenomics are noncoding regulatory molecules, an extensive system representing more than 80% of the human transcriptome. To underline their importance, a recent large-scale study, screening a multitude of cell lines, has discovered the significance of long noncoding RNA (lncRNA) in anticancer treatments. LncRNA transcriptome appears to be as important as the protein-coding transcriptome in the analysis of drug responses to hundreds of compounds [106].

LncRNAs affect drug metabolism and disposition through regulating phase I and phase II biotransformation enzymes and ABC transporters [107]. LncRNAs have been found to regulate the expression of *ABCB1*, *ABCC1* and *ABCG2* and modulate therapy responses in breast cancer, lung cancer and gastric cancer (reviewed in [108]). As a further example, lncRNAs have been implicated in the chemoresistance of triple-negative breast cancer (TNBC). In addition to their effects on drug metabolism and efflux, they may also facilitate TNBC’s escape from apoptosis and evasion of the immune system (reviewed in Kansara et al. [109]). 

LncRNA *MALAT1* positively regulates the expression of *ABCB1* and *ABCC1* transporters via the activation of the *STAT3* transcription factor [110]. In a French study using real-time PCR and reverse phase protein array (RPPA) techniques, *MALAT1* was overexpressed in mammary tumors. Interestingly, an alternatively spliced transcript *Δsv-MALAT1* appeared as an independent factor for a good prognosis in breast cancer patients [111]. Next, LINC00963 was found upregulated in head and neck carcinomas. As was further shown, a knockdown of LINC00963 resulted in a decreased invasion, colony formation and self-renewal via the regulation of *ABCB5* [112]. A knockout of another lncRNA *HNF1α-AS1* decreased the mRNA expression of various CYPs including *CYP2B6, 2C8, 2C9, 2C19, 2E1,* and *3A4,* while the knockout of *HNF4α-AS1* increased the CYP mRNA levels in HepaRG cells in vitro. CYPs are regulated by *HNF1α-AS1* and *HNF4α-AS1* via nuclear receptors PXR and CAR [113,114]. Noteworthy are also the roles of MDR1-targeting lncRNA *HOTAIR* and of other lncRNAs in the development of cisplatin chemoresistance in various cancers as was recently reviewed by Hu et al. [115]. A number of lncRNAs were markedly differentially expressed in 33 Czech hepatocellular carcinoma patients between patients with downregulated CYP expression vs. patients with normal CYP levels [116].

Thus, lncRNAs are a new and rapidly expanding area of pharmacogenomics. Despite its relatively short history, the accumulating evidence points to a highly valuable source for predicting cancer responsiveness in the future, especially if combined with gene variant and expression data.

## 6. Conclusions

The emerging role of pharmacogenomics in the NGS era provides valuable tools for determining inter-individual variations and should be soon taken into account in drug prescribing. The sources of “Big Data” in public databases originating from large genotyping projects might become a part of medical records. 

Promising examples of clinical applications of pharmacogenomics may be seen in the area of targeted therapy of selected cancers with TKIs and the list is envisaged to expand soon. Historically, research of ABCs and CYPs in pharmacology and oncology was directed at their expression and germline genetic variation. More focus on somatic variations is needed. Databases like TCGA are vast repositories of tens of thousands of whole exome or genome sequences of tumor and germline DNA from patients with practically all types of cancers. A substantial portion of this potential still remains unexplored and unexploited. Rare variants in pharmacogenes and epigenetic features such as lncRNA represent another treasury of pharmacogenetic variation in a population, apart from the long-established hereditary germline polymorphisms and somatic variants in tumors. In addition, CNVs represent another piece of this puzzle. On the other hand, it has to be kept in mind that pharmacogenomics does not provide information about the post-translational modifications of encoded proteins and thus the role of this aspect for cancer therapy also needs further assessment.

Although the pharmacogenomics data on CYPs and ABCs reviewed above represent the forefront of experimental oncology, the lack of a thorough validation of published observations and standardization of new research studies in a way similar to clinical trials impedes further progress in clinical applications. Future studies should bear in mind that cancer therapy is an extremely complex process. Clinical data and all other interacting factors, for example, lifestyle and the resulting drug–drug and drug–dietary interactions, should be included as well as the real-time monitoring of patients’ response to therapy which sometimes exceeds a decade, e.g., in breast cancer. Truly precise personalized medicine may be enabled only after considering the aforementioned facts, robust replication of contemporary findings and methodological standardization, careful exploration of emerging NGS data and sophisticated utilization of integrative approaches.

## Figures and Tables

**Table 1:** Official U.S. Food and Drug Administration (FDA) pharmacogenomic biomarkers in CYPs for anticancer drugs.

Gene	Drug	Pharmacogenomics Level	Recommendation	Disease
*CYP1A2*	rucaparib	informative	no difference in PK among metabolizer phenotypes	epithelial ovarian cancer, fallopian tube, primary peritoneal cancer
*CYP2C9*	erdafitinib	actionable	lower dose for CYP2C9*3/*3 genotype	urothelial carcinoma
*CYP2D6*	gefitinib	actionable	ADR risk for poor metabolizers	non-small cell lung cancer
*CYP2D6*	rucaparib	informative	no difference in PK among metabolizer phenotypes	epithelial ovarian cancer, fallopian tube, primary peritoneal cancer
*CYP2D6*	tamoxifen	actionable	poor metabolizers have lower endoxifen concentrations	breast cancer

PK—pharmacokinetics; ADR—adverse drug reactions.

**Table 3:** Information about germline copy number variations in ABC and CYP genes in Genome Aggregation Database (gnomAD) database.

Gene	Variant ID	Consequence	Class	Size	Allele Count ^1^
*ABCA3*	DEL_16_151377	intronic	deletion	122 bp	1
	DEL_16_151378	intronic	deletion	824 bp	18
	DEL_16_151379	intronic	deletion	434 bp	208
	DEL_16_151381	intronic	deletion	477 bp	1
	INS_16_98759	intronic	insertion	279 bp	1
*ABCD3*	INV_1_34	inversion span	inversion	19.4 Mb	1
	INV_1_35	inversion span	inversion	652 kb	1
	DUP_1_1758	copy gain	duplication	321 kb	2
	DEL_1_6074	intronic	deletion	1.04 kb	1
	DEL_1_6075	intronic	deletion	6.0 kb	1
	DEL_1_6076	intronic	deletion	90 bp	6408
	INS_1_3772	intronic	insertion	281 bp	2
	DUP_1_1759	partial duplication	duplication	97 bp	3
*ABCE1*	CPX_4_1427	inversion span	complex	87.0 Mb	1
	DEL_4_50987	intronic	deletion	485 bp	1
*CYP2D6*	MCNV_22_1026	MCNV overlap	multi CNV	12.2 kb	2881
	DEL_22_182964	intronic	deletion	105 bp	1
*CYP4A11*	INV_1_10	inversion span	inversion	58.3 Mb	1
	DUP_1_1171	copy gain	duplication	118 kb	3
	DUP_1_1173	partial duplication	duplication	82.0 kb	2
	DUP_1_1174	copy gain	duplication	201 kb	6
*CYP17A1*	CPX_10_3184	inversion span	complex	106 Mb	1
	INV_10_527	inversion span	inversion	15.6 Mb	1
	DUP_10_30635	partial duplication	duplication	14.4 kb	1

^1^ Out of 15,708 whole-genome sequences from unrelated individuals. MCNV—multiCNV.
